# Variation in nuclear genome size in a freshwater snail model system featuring a recent whole-genome duplication

**DOI:** 10.1098/rsos.250171

**Published:** 2025-06-04

**Authors:** Maurine Neiman, Maria Pichler, Martin Haase, Dunja K. Lamatsch

**Affiliations:** ^1^ Department of Biology, University of Iowa, Iowa City, IA, USA; ^2^ Research Institute of Limnology, University of Innsbruck, Mondsee, Austria; ^3^ Zoologisches Institut und Museum, Ernst-Moritz-Arndt Universitat Greifswald Mathematisch-Naturwissenschaftliche Fakultat, Greifswald, Mecklenburg-Vorpommern, Germany

**Keywords:** polyploid, flow cytometry, diploid, parthenogenetic, mollusc, snail

## Abstract

Conspecifics often share—or are assumed to share—nuclear genome characteristics like nucleotide composition and genome size. These fundamental aspects of the nuclear genome can themselves be the object of natural selection. We here provide the first high-quality direct measurements of nuclear genome DNA content in a representative diverse sample of *Potamopyrgus antipodarum*, an Aotearoa New Zealand freshwater snail that is a textbook example of the maintenance of sexual reproduction in nature and is invasive worldwide. We used propidium-iodide-based flow cytometry to characterize nuclear DNA content and its variation in nearly 100 *P. antipodarum* from multiple populations representing both sexual and asexual individuals. We also estimated nuclear DNA content in multiple *P. estuarinus*, a closely related obligately sexual species. These data confirmed and extended earlier lines of evidence for polyploidy and variable genome size within asexual *P. antipodarum* and provided the first direct demonstration of distinctly higher nuclear genome content in diploid (sexual) *P. antipodarum* relative to diploid sexual *P. estuarinus*. Together, these results are consistent with genomic evidence for a recent whole-genome duplication (WGD) and subsequent and in-process rediploidization in *P. antipodarum*, setting the stage for use of *Potamopyrgus* as a model for WGD and its consequences.

## 1. Introduction

The size, structure and composition of the nuclear genome are together perhaps the defining trait of any given unique taxon, and each plays central but distinct roles in shaping evolutionary trajectories and organismal ecology (e.g. [1–6]. While whole-genome sequencing efforts can definitively establish nucleotide composition, the physical size of the genome (the amount of DNA in an unreplicated somatic nucleus, here ‘nuclear DNA content’) and genomic structure (e.g. karyotype) cannot always be rigorously and accurately characterized by even high-quality nuclear genome sequence assemblies [7,8]. This issue is exacerbated by common genomic features like repetitive content and high levels of heterochromatism, both of which are often themselves associated with relatively large genomes [9] Because nuclear DNA content can differ between closely related taxa and even conspecifics ([10]; e.g. [11–14]; also see https://www.genomesize.com/), one needs to estimate nuclear DNA content in a representative sample of conspecifics to provide an accurate picture of nuclear genome size (including potential for variation) for any given species.

A simple and relatively old technology, flow cytometry [7,8], when appropriate intercalating fluorochromes (e.g. propidium iodide, PI) and internal standardization are used ([15]; e.g. [16]), is still the gold standard for providing accurate and direct estimates of nuclear genome DNA content [10]. Here, we use PI-based flow cytometry to provide definitive estimates of nuclear genome DNA content across ploidy levels in an Aotearoa New Zealand snail species, *Potamopyrgus antipodarum*, which has risen to prominence because of notably wide within- and across-population variation in reproductive mode and ploidy level [12,17–19], and is invasive worldwide [20]. We also use flow cytometry to provide nuclear DNA content estimates for an obligately sexual and diploid congener, *P. estuarinus*.

These new data on nuclear DNA content in *Potamopyrgus* are important in providing an accurate picture of a fundamentally important trait for an important model system. Our results also provide a distinct line of evidence in support of the hypothesis that *P. antipodarum* appears to have experienced a very recent whole-genome duplication (WGD) that might be in turn connected to its unusually frequent transitions to asexual reproduction [21–23]. With respect to the WGD, accurate characterization of genome size in *P. antipodarum* and a congener that reasonably represents the ‘ancestral’ (pre-duplication) nuclear genomic DNA content [22] is a critical piece of understanding the causes and consequences of this recent dramatic change in genome structure.

## 2. Methods: flow cytometry and estimation of nuclear DNA content

We used flow cytometry to measure nuclear DNA content following the detergent-trypsin method and PI staining approach detailed in [24], with minor modifications. Details regarding the specific standards used and when flow cytometry was conducted for each of our samples can be found in electronic supplementary material, table S1. We quantified nuclear genome DNA content in 99 *P*. *antipodarum* and 10 *P*. *estuarinus*. The *P. antipodarum* we used were either collected from natural populations in New Zealand (*n* = 56, 9 sites) or an invasive population in Austria (*n* = 5, 1 site), sourced from laboratory-cultured lineages descended from single females originally collected from New Zealand lakes (*n* = 32, 8 lineages), or sampled from diverse and uncharacterized tanks of *P. antipodarum* kept in the Neiman lab (*n* = 6, 3 tanks) (table 1).

**Table 1.** Snails and sources.

species	source	coordinates	type	*n*	mean nuclear genome content (pg; *Drosophila*) (s.d.)	% 2*n*	mean 2*n* nuclear genome content (pg) (s.d.)	% 3*n*	mean 3*n* nuclear genome content (pg) (s.d.)	% 4*n*	mean 4*n* nuclear genome content (pg) (s.d.)
*P. antipodarum*	Boyd Creek	45.0807° S, 167.5659° E	field	6	2.716 (0.052)	0	NA	100	2.716 (0.052)	0	NA
*P. antipodarum*	Buller Gorge	41.5006° S, 171.4002° E	field	4	2.695 (0.021)	0	NA	100	2.695 (0.021)	0	NA
*P. antipodarum*	Coromandel Peninsula	36.5120° S, 175.3409° E	field	12	1.530 (0.043)	100	1.530 (0.043)	0	NA	0	NA
*P. antipodarum*	diverse triploid mix^a^	NA	lab tank	1	2.830 (NA)	0	NA	100	2.830 (NA)	0	NA
*P. antipodarum*	Franklin Road	37.2746° S, 175.4649° E	field	6	2.660 (0.045)	0	NA	100	2.660 (0.045)	0	NA
*P. antipodarum*	Gunn-06	44.8759° S, 168.0903° E	lab lineage	4	3.641 (0.080)	0	NA	0	NA	100	3.641 (0.080)
*P. antipodarum*	Gunn-07	44.8759° S, 168.0903° E	lab lineage	4	3.412 (0.458)	0	NA	25	2.727 (NA)	75	3.641 (0.045)
*P. antipodarum*	Gunn-10	44.8759° S, 168.0903° E	lab lineage	3	3.626 (0.029)	0	NA	0	NA	100	3.626 (0.029)
*P. antipodarum*	Karangahake Gorge	37.2546° S, 175.4336° E	field	6	1.741 (0.051)	100	1.741 (0.051)	0	NA	0	NA
*P. antipodarum*	Lady/Ianthe^b^	42.3558° S, 171.3425° E (Lady)/43.0500° S, 170.6167° E (Ianthe)	field	5	1.809 (0.054)	100	1.809 (0.054)	0	NA	0	NA
*P. antipodarum*	Mapourika 18-7	43.3180° S, 170.2042° E	lab lineage	6	2.137 (0.493)	66.7	1.819 (0.011)	33.3	2.772 (0.045)	0	NA
*P. antipodarum*	Miti Miti	34.3544° S, 172.5559° E	field	6	2.654 (0.069)	0	NA	100	2.654 (0.069)	0	NA
*P. antipodarum*	Mondsee	47.8210° N, 13.3752° E	field	5	2.531 (0.055)	0	NA	100	2.531 (0.055)	0	NA
*P. antipodarum*	Niagara Falls	46.3541° S, 169.0805° E	field	5	2.774 (0.069)	0	NA	100	2.774 (0.069)	0	NA
*P. antipodarum*	outbred sexual^c^	NA	lab tank	1	1.897 (NA)	100	1.897 (NA)	0	NA	0	NA
*P. antipodarum*	Poerua-72	42.7048° S, 171.4952° E	lab lineage	3	3.788 (0.056)	0	NA	0	NA	100	3.788 (0.056)
*P. antipodarum*	Poerua-92	42.7048° S, 171.4952° E	lab lineage	3	2.716 (0.016)	0	NA	100	2.716 (0.016)	0	NA
*P. antipodarum*	Selfe 18-1	43.2403° S, 171.5199° E	lab lineage	6	1.989 (0.361)	83.7	1.841 (0.041)	16.7	2.721 (NA)	0	NA
*P. antipodarum*	Selfe 18-46	43.2403° S, 171.5199° E	lab lineage	3	2.867 (0.112)	0	NA	100	2.867 (0.111)	0	NA
*P. antipodarum*	Te Anau	45.2138° S, 167.7500° E	field	6	2.995 (0.431)	0	NA	83.7	2.821 (0.069)	16.7	3.866 (NA)
*P. antipodarum*	West Coast sexual^d^	NA	lab tank	4	1.758 (0.044)	100	1.758 (0.044)	0	NA	0	NA
*P. estuarinus*	Waikuku	43.2870° S 172.7160° E	field	10	1.370 (0.054)	100	1.370 (0.054)	0	NA	0	NA

^a^ Snails from this source were sampled from a tank harbouring descendents of asexual individuals collected from various South Island New Zealand lakes in 2009 and 2010.

^b^ Snails from this source were sampled from a tank harbouring a diverse mix of sexual individuals collected from New Zealand lakes Lady and Ianthe in 2018.

^c^ Snails from this source were sampled from a tank harbouring descendents of sexual individuals collected from multiple South Island New Zealand lakes in 2018 and 2020.

^d^ Snails from this source were sampled from a tank harbouring descendents of sexual individuals collected from multiple South Island New Zealand lakes in 2015 and 2016.

We first followed standard procedures (e.g. [25]) to determine whether a penis was present (male) or absent (female) and thus sex each snail. Next, we dissected away the head tissue of each snail and snap-froze the tissue in liquid nitrogen, while the body tissue was fixed for chromosome preparation in Carnoy fixative. The head was homogenized in a citrate buffer (3.4 mM trisodium citrate dihydrate, Nonidet P40 at 0.1% v/v, 1.5 mM sperminetetrahydrochloride, 0.5 mM trishydroxymethylaminomethane, pH 7.6), and was then transferred to 750 μl stock solution in a 1 ml Kimble Dounce tissue grinder (Sigma Aldrich) and homogenized on ice with 20 strokes using the ‘tight’ pestle of the homogenizer. As an internal standard of known genome size, we used the fruit fly *Drosophila melanogaster* (genotype ISO-1, diploid nuclear DNA content: 0.350 pg and genotype DGRP-208, diploid nuclear DNA content: 0.321 pg [26]) and/or self-collected female chicken (*Gallus gallus*) red blood cells (diploid nuclear DNA content = 2.50 pg [27]). The same procedure used to prepare and run samples for snail flow cytometry was applied to head tissue dissected from each of 10 *D. melanogaster* females and to the 200 µl of chicken red blood cells (CRBC).

**Figure 1.**
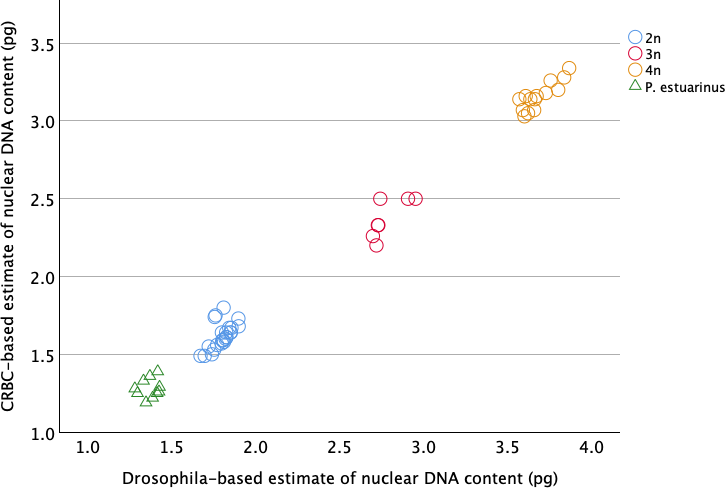
Scatterplot comparing the *D. melanogaster*-based estimates of nuclear DNA content in *Potamopyrgus* relative to the chicken red blood cell-based measurements for the same samples. Open circles represent *P. antipodarum* and the triangles represent *P. estuarinus*. Ploidy assignments reflect the position of each snail in the trimodal distribution shown in figure 2.

**Figure 2.**
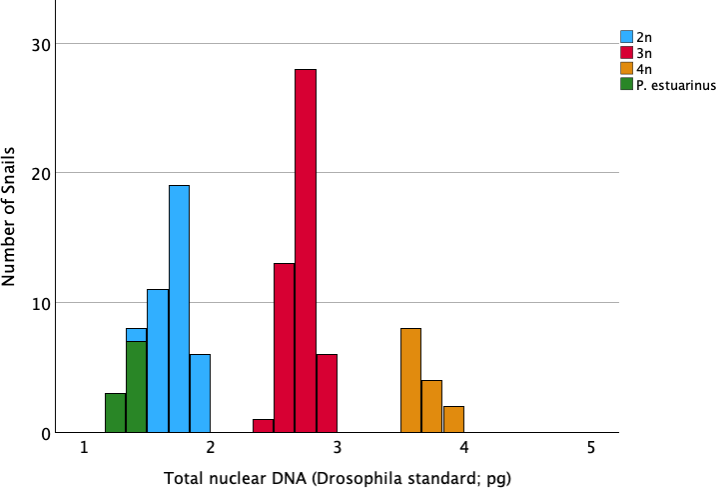
Histogram of genome size estimates across all 109 *Potamopyrgus*, colour-coded according to species status (*P. antipodarum* versus *P. estuarinus*) and ploidy (*P. antipodarum*), the latter assigned through position of data from each snail within the trimodal histogram for *P. antipodarum*. There is no evidence for ploidy variation within the diploid and obligately sexual *P. estuarinus*.

**Figure 3.**
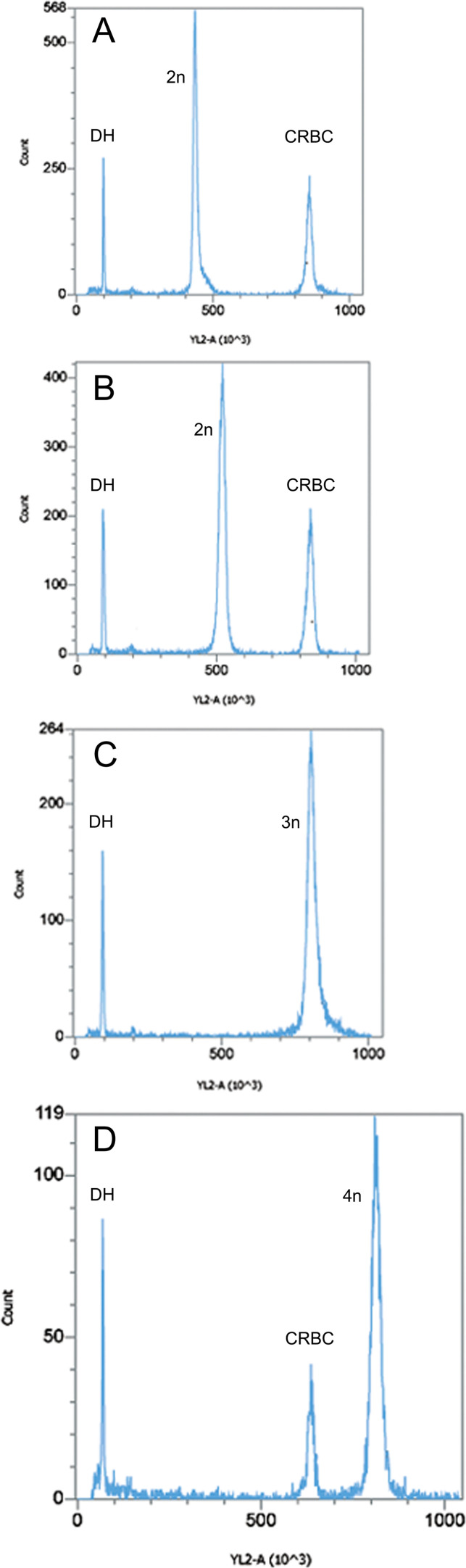
Examples of PI fluorescence peaks for (A) exemplar *P. estuarinus*, (B) diploid *P. antipodarum*, (C) triploid *P. antipodarum*, (D) tetraploid *P. antipodarum* (different scale, to accommodate higher nuclear DNA content). Each example also includes the standards spiked into that particular run, with ‘DH‘ peaks representing *D. melanogaster* head standards and 'CRBC' representing chicken red blood cell standards.

A Pearson’s correlation analysis demonstrated that genome size estimates for the 55 samples for which we used both *D. melanogaster* and CRBC standards to estimate nuclear genome DNA content were virtually identical (*r* = 0.996, *p* < 0.001; figure 1). This high correspondence between CRBC and *D. melanogaster* is especially important in light of the fact that the small size of the fly heads translates into higher CVs for fluorescence (see electronic supplementary material, table S1). We nevertheless prefer to use only *D. melanogaster* standards for our genome content estimates (*n* = 54 samples) because, unlike CRBC (2.5 pg), the nuclear DNA content of *D. melanogaster* (<<1 pg) is much lower than for even diploid *P. antipodarum* or *P. estuarinus* (figures 1–3), facilitating the differentiation of snail versus standard.

Large debris was removed by filtration through a 40 μm mesh nylon sieve before measurement. After the addition of 100 μl of 0.021% trypsin (dissolved in stock solution), the sample was incubated for exactly 10 min at 37°C. To stop the digestion, 75 µl of 0.25% trypsin inhibitor was added (this solution also included 0.05% RNAse A), and the samples were incubated for another 10 min at 37°C. Finally, samples were stained with PI at a concentration of 50 μg ml^−1^. Stained samples were kept overnight on ice in the dark. Flow cytometric analysis was performed on the next day on an Attune NxT^®^ acoustic focusing cytometer (Thermo Fisher) with an excitation wavelength of 561 nm and a custom-made 590−650 nm bandpass filter (yellow, YL-2) for detection of PI fluorescence. To exclude doublets (i.e. nuclei that pass the detector too close together, thus being recorded as a single ‘event’), we used YL2-A versus YL2-H gating. We first ran the homogenized and stained snail cells on their own to check quality (i.e. visual assessment of the variance around the snail fluorescence peak) without the standard. If the snail sample appeared to be of acceptable quality (i.e. this fluorescence peak is relatively narrow, suggesting low variance of fluorescence estimates), we then mixed the homogenized and stained cells from the snail and the standard and ran the sample again to provide a standard-calibrated estimate of nuclear genome content in the snail. We provide examples of these fluorescence peaks for diploid, triploid and tetraploid *P. antipodarum* and for *P. estuarinus* in figure 3.

Coefficients of variance (CV) of individual fluorescent peaks had a mean of 2.02% (s.d. ± 0.77%) for snails, 1.48% (s.d. ± 0.18%) for CRBC, and 4.07% (s.d. ± 1.59%) for *D. melanogaster*. Conversion from picograms of DNA to base pairs was made following 1 pg = 978 Mbp [26]. Following earlier flow cytometry-based studies in *P. antipodarum* [12,19], we visualized these nuclear genome DNA content data in the form of a histogram based on pg per nucleus. We thus assigned diploid, triploid or tetraploid status for each *P. antipodarum* depending on where snail DNA content fit into the trimodal distribution of data (figure 2).

## 3. Results and discussion

We used propidium iodide-based flow cytometry to estimate nuclear genome DNA content for 99 *P. antipodarum*, a unique and prominent model system in ecology and evolution, and 10 *P*. *estuarinus*. This effort constitutes the first of which we are aware to use this gold-standard method for nuclear genome size quantification for a large sample of *P. antipodarum* from different populations, along with a congener often used for comparative studies. Such data are useful in themselves for any model system, but are of especially critical importance in providing an independent line of evidence suggesting that *P. antipodarum* has experienced a very recent whole-genome duplication ([21]; also see [22]).

As expected from earlier flow cytometry [12,19,28], population genetic [19,29,30] and cytogenetic data [18,31], the distribution of nuclear DNA content within *P. antipodarum* was trimodal, corresponding to diploid, triploid and tetraploid individuals (figure 2). Accordingly, we assigned each *P. antipodarum* individual to a ploidy level depending on its placement in one of the three nuclear DNA content peaks: diploid (mean nuclear DNA content = 1.71 pg ± 0.14 s.d., *n* = 37), triploid (mean nuclear DNA content = 2.71 pg ± 0.10 s.d., *n* = 48; 1.59 × versus diploids), or tetraploid (mean nuclear DNA content = 3.69 pg ± 0.10 s.d., *n* = 14; 2.16 × versus diploids and 1.36 × versus triploids) (figures 1 and 2). The mean nuclear DNA content for the 10 *P*. *estuarinus* was 1.370 pg per nucleus (s.d. ± 0.05), 80% of that of diploid *P. antipodarum* (figures 1 and 2). Our PI-based estimate of nuclear genome DNA content in *P. estuarinus* is the first of which we are aware, but it is aligned with previous genomic sequencing-centred approaches to genome size estimation in this species ([22]; electronic supplementary material, table S2). The distinctly higher nuclear DNA content of *P. antipodarum* relative to *P. estuarinus* provides yet another line of evidence pointing to a very recent whole-genome duplication in the former [21–23].

**Figure 4.**
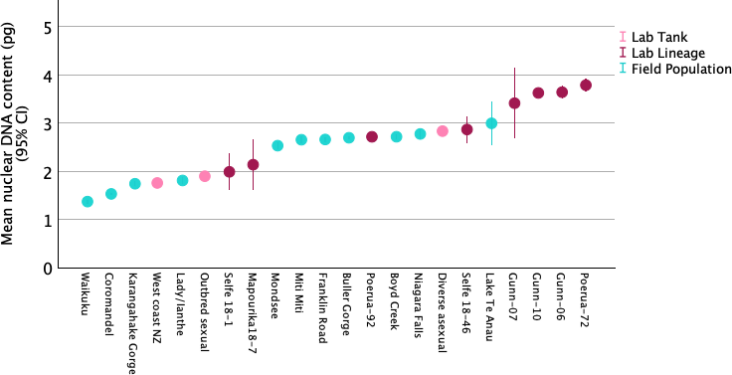
Comparisons across mean nuclear DNA content from snails collected from natural populations and laboratory tanks and lineages, rank ordered from low to high. The especially wide 95% CI around the means for Selfe 18-1, Mapourika 18-7, Gunn-07 and Lake Te Anau reflect ploidy polymorphism within the samples; all other samples were only represented by one ploidy level. Diploid or predominately diploid sources ranged from Waikuku (smallest mean nuclear DNA content) to Selfe 18-1 and Mapourika 18-7 (lineages with the highest mean nuclear DNA content for a ‘diploid’, but both of which unexpectedly also harboured triploid individuals), triploid or predominantly triploid samples ranged from Mondsee (smallest mean nuclear DNA content) to Te Anau (including one unexpected tetraploid individual), and tetraploid or predominantly tetraploid samples ranged from Gunn-07 (including one unexpected triploid individual) to Poerua 72.

**Figure 5.**
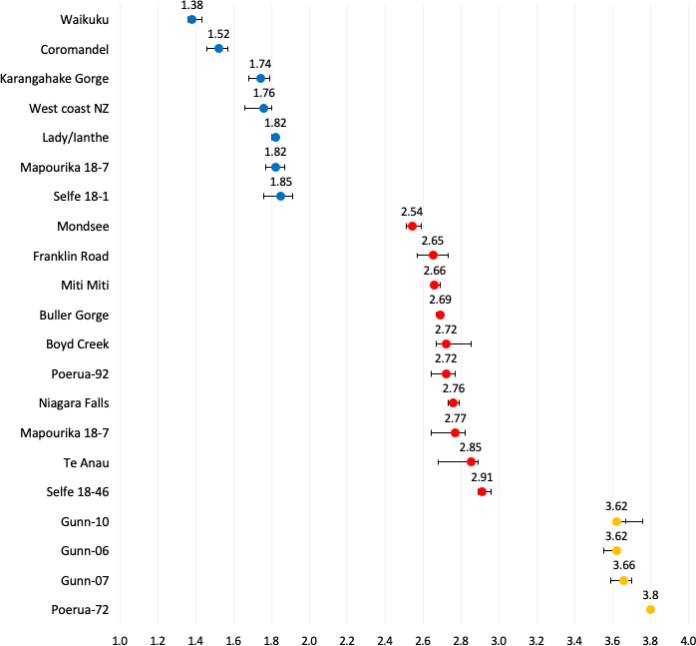
Comparison of median nuclear DNA content (pg; *x* axis) across source populations, tanks and lineages within ploidy levels. Samples are rank-ordered from top to bottom by median pg within ploidy level from low to high (electronic supplementary material, table S3). Diploids are blue, triploids are red, and tetraploids are yellow. Only snail sources represented by more than one individual per ploidy level are included (see table 1). ‘Waikuku’ in the 2*n* panel is the *P. estuarinus* sample. Error bars represent 95% CIs for median nuclear DNA content generated by 884−1000 bootstrap samples as executed within IBM SPSS (electronic supplementary material, table S3).

In light of 4′,6-diamidino-2-phenylindole (DAPI)-based flow cytometry data that indicated wide variation in nuclear DNA content both across populations and within ploidies in *P. antipodarum* [12] and similar variation within and among asexual triploid *P. antipodarum* lineages from a single New Zealand lake [28], we also compared nuclear DNA content across the 11 natural collections and 8 laboratory lineages in our *P. antipodarum* dataset. Similar to these earlier data, our PI-based estimates reveal variation within natural populations across ploidy levels (Te Anau, with five triploids and one tetraploid in our sample of six snails) as well as across snail sources within ploidy levels (figures 4 and 5). We also see two cases of ploidy variation *within* laboratory lineages: there was one triploid snail in the otherwise diploid Selfe 18-1 lineage and one triploid snail in the otherwise tetraploid Gunn-07 lineage.

We used a bootstrapping approach as implemented within IBM SPSS v. 29 software to compare 95% CIs across median nuclear genome content within ploidy levels and across snail sources (electronic supplementary material, table S3). This analysis revealed that some *P. antipodarum* harboured significantly different nuclear DNA content even within ploidy levels, based on non-overlapping 95% CI around medians (figure 5). For example, Coromandel Peninsula 2*n* snails have significantly lower nuclear DNA content than all other 2*n P*. *antipodarum*, but still significantly higher nuclear DNA content than *P. estuarinus*, and 4*n* Poerua-72 snails have significantly higher nuclear DNA content than at least two of the three 4*n* lineages from lake Gunn. These outcomes are consistent with and extend earlier reports of such variation in *P. antipodarum* [12,28], which is in turn potentially a consequence of variable chromosome number ([18,31,32] and/or variation in the extent of accumulation of an approximately 18 kb tandemly repeated block of histone and ribosomal DNA genes [31] or other repetitive elements (e.g. [33]). Definitive assessment of these non-mutually exclusive hypotheses will require cytogenetic and genomic analysis of individuals with the same assigned ploidy level but differing in nuclear genome DNA content.

The distinctly lower nuclear genome content of the snails from the Coromandel Peninsula relative to other presumably diploid and sexual *P. antipodarum* (figures 4 and 5) prompted us to consider whether these individuals might instead represent a distinct species. We followed Haase [34], who used careful morphological observation coupled with assessment of mitochondrial DNA sequence divergence relative to other *Potamopyrgus* taxa to demonstrate the presence of cryptic *Potamopyrgus* species in New Zealand in his 2008 revision of the radiation of tateid (at the time attributed to Hydrobiidae; see [35]) gastropods in New Zealand. We dissected each of three Coromandel Peninsula males and females to characterize genital anatomies and assess oviparous versus ovoviviparous status. We also sequenced a 503 bp fragment of mitochondrial *cytochrome b* (see [36] for primers and polymerase chain reaction (PCR) conditions) in an additional seven Coromandel Peninsula individuals to determine whether even phenotypically similar individuals might differ genetically.

Genital anatomy is diagnostic of species in *Potamopyrgus* [34], and the fact that we did not detect any such anatomical differences with respect to *P. antipodarum* for the Coromandel Peninsula snails and that these snails were ovoviviparous indicates that the Coromandel Peninsula snails are *P. antipodarum*. This conclusion is bolstered by the mtDNA data: the two haplotypes detected in the seven snails (identical in six individuals, differing at one position for one snail) were 98.21–99.40% (Genbank accession PQ962506) and 98.21–99.60% (PQ962507) identical to the first 30 hits of *P. antipodarum* in a BLAST search against the core nucleotide database of GenBank (21 January 2025; https://blast.ncbi.nlm.nih.gov/Blast.cgi). The closest hit between *P. antipodarum* and another *Potamopyrgus* species (*P. kaitunuparaoa*) had only 94.01% pairwise identity. Thus, neither genital anatomy, mode of egg production, nor mitochondrial sequence information indicated that the population from the Coromandel Peninsula, despite its distinctly smaller nuclear genome DNA content, represented a species other than *P. antipodarum*.

The discovery of a *P. antipodarum* population with smaller nuclear genome DNA content than what appears to be the majority of its otherwise similar diploid sexual counterparts sets the stage for follow-up work more directly evaluating the causes and consequences of the extensive genome size variation, both within and across ploidy levels, in this species. Especially productive next steps might include sequencing of nuclear markers that will allow us to verify or refute the tentative conclusion regarding the assignment of the Coromandel Peninsula snails as members of *P. antipodarum*, high-coverage genomic sequencing and cytogenetic characterization of Coromandel Peninsula snails to assess the extent of representation (or lack thereof) by repetitive elements (following [31]), and crossing experiments between Coromandel Peninsula snails and diploid sexual *P. antipodarum* with the more ‘standard’ higher nuclear DNA content followed by genomic and cytogenetic characterization of offspring.

## 4. Summary, conclusions and next steps

We used PI-based quantification of nuclear genome DNA content in a diverse sample of *P. antipodarum* and multiple *P. estuarinus* to provide the first rigorous and comprehensive characterization of this fundamental genomic trait in a genus known around the world as a model system for the evolution of sex and host–parasite interactions and as a biological invader. These analyses validated and qualitatively extended previously established evidence for polyploidy and variable nuclear genome DNA content *within* ploidy levels in *P. antipodarum*. This work also provides a distinct line of support for a recent WGD in the lineage leading to *P. antipodarum* after its divergence from the shared common ancestor with *P. estuarinus*. Together, these data provide a concrete example of the importance of surveying a large and diverse sample of individuals with respect to accurately assessing intraspecific variation in nuclear genome DNA content and set the stage for the use of *Potamopyrgus* as a powerful system to explore questions regarding the causes and consequences of genome size variation and the aftermath of a recent WGD.

## Data Availability

All snail, source and flow cytometry data used for the paper are available within 'electronic supplementary material, table 1' [37]. The unique mitochondrial haplotypes we generated for the Coromandel Peninsula snails are available in Genbank (accession nos.: PQ962506, PQ962507).

## Dedication

This paper is dedicated to JJ Neiman-Brown, who made snail collections and a guest professorship in Austria such a joy. May his memory be a blessing.
